# Alexandrite Laser-Induced Choroidal Neovascularization Successfully Treated With Aflibercept

**DOI:** 10.7759/cureus.8294

**Published:** 2020-05-26

**Authors:** Asterios Diafas, Despoina Stampouli, Anna Dastiridou, Sofia Androudi, Periklis Brazitikos

**Affiliations:** 1 Ophthalmology, Papageorgiou General Hospital / Aristotle University of Thessaloniki, Thessaloniki, GRC; 2 Ophthalmology, General Hospital of Xanthi, Xanthi, GRC; 3 Ophthalmology, University of Thessaly, Larissa, GRC

**Keywords:** choroidal neovascularization, hair removal, alexandrite laser, retinal injury, aflibercept

## Abstract

A 33-year-old female was referred to the ophthalmology department after an accidental eye injury to her right eye during a hair removal session using alexandrite laser. Although she initially experienced no symptoms, when re-examined one and a half months later the best-corrected visual acuity (BCVA) of the affected eye was 20/40 secondary to choroidal neovascularization confirmed by fluorescein angiography (FA) and optical coherence tomography (OCT). Intravitreal anti-vascular endothelial growth factor (VEGF) therapy (three monthly injections of aflibercept) led to complete regression of the neovascularization and functional recovery which was maintained at one-year follow-up post original injury.

## Introduction

The retina is the most vulnerable tissue to laser-derived radiation. The severity of any consequent injury and its effect on visual outcome depend on a variety of laser-specific and eye-dependent factors [1]. The damage caused by a laser depends on its wavelength, the duration of pulse, the amount of energy delivered and damage location on the retina (its proximity to the fovea). Laser-induced retinal injury has been reported to be associated with vitreous, sub-retinal and chorioretinal haemorrhage, retinal edema, epiretinal membrane formation, retinal photoreceptor disruption and macular hole [2,3]. It is rarely reported that such injury can cause choroidal neovascularization [4]. In recent years, we have seen an increase in the use of laser systems, with alexandrite laser being the most widely used, in the field of aesthetics for primarily hair removal procedures. We present the development and management of sub-retinal neovascularization following macular photic trauma after accidental exposure to alexandrite laser.

## Case presentation

A 33-year-old female was referred to our clinic, after accidental exposure to alexandrite laser (750 nm) during a hair removal session (without the use of protective goggles). Pulse duration was 3 ms and energy density was approximately 10 J/cm^2^. The patient did not report any symptoms during her first ophthalmologic examination and her best-corrected visual acuity (BCVA) was 20/20 in both eyes. Anterior segment examination was unremarkable and intraocular pressure was 13 mmHg in both eyes. However, fundus examination of the right eye revealed a spot on the parafoveal region presumably caused by laser-induced burn. Optical coherence tomography (OCT) revealed focal disruption of inner/outer segment junction of photoreceptor and outer plexiform/nuclear and retinal pigment epithelium (RPE) focal deformations. Topical steroid eye drops and non-steroid anti-inflammatory eye drops were prescribed four times and once daily, respectively. Tapering doses of steroid eye drops were administered during a four-week period.

**Figure 1:**
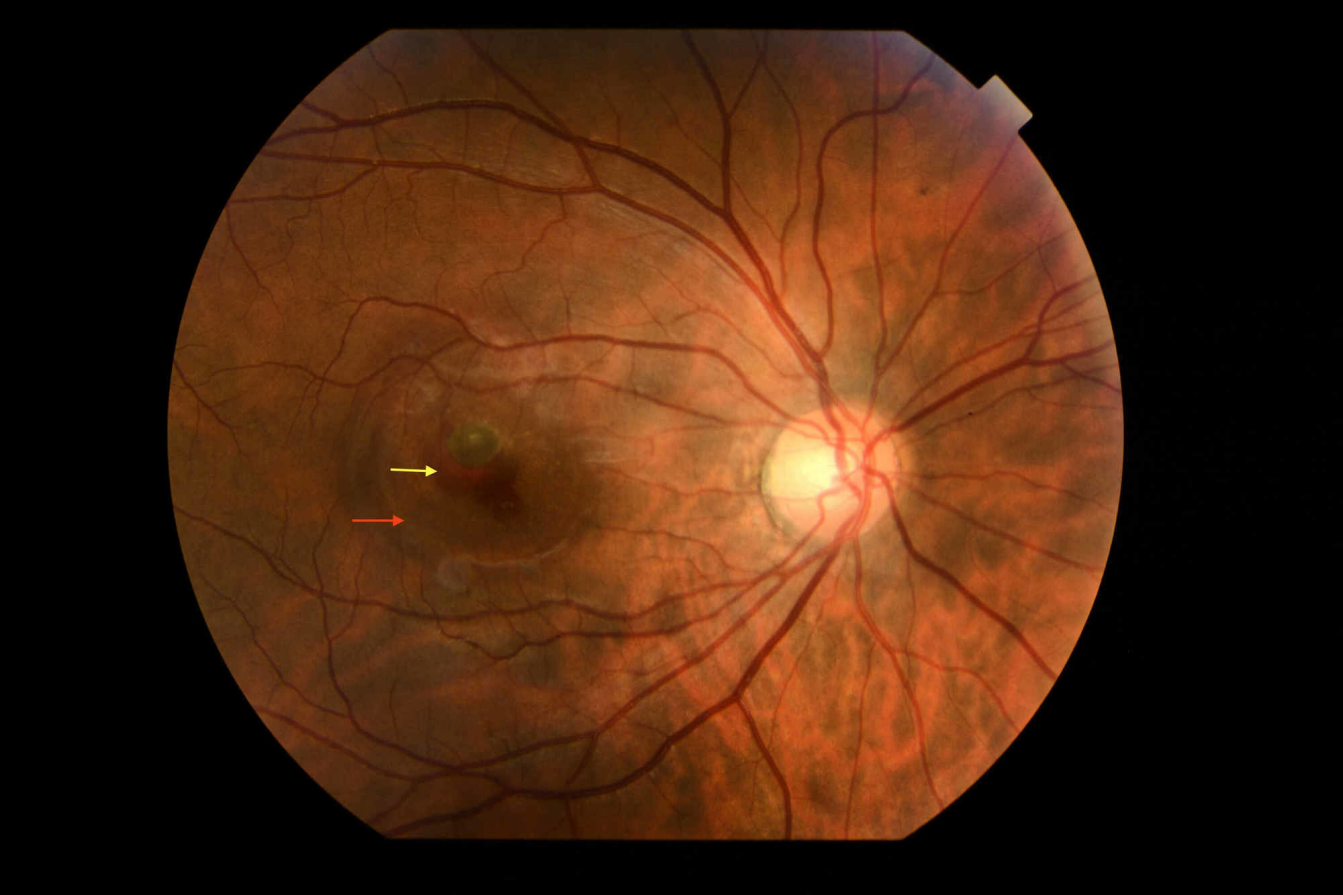
Fundus photography showing sub-retinal haemorrhage (yellow arrow) and edema (red arrow)

**Figure 2:**
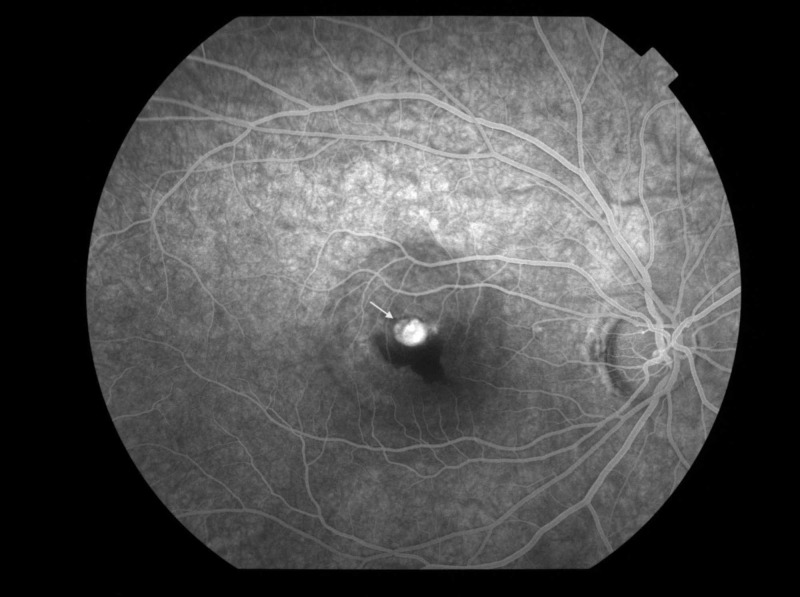
Fluorescein angiography (FA) photography showing hyperfluorescence with leakage (arrow) in the late phase suggestive of a choroidal neovascular membrane (2nd visit - a month and a half after injury)

A month and a half later the patient complained of blurred and reduced vision from her right eye. Upon clinical examination, BCVA in the right eye was 20/40, the anterior segment was normal and the intraocular pressure was 14 mmHg. Clinical examination in the left eye was unremarkable. Fundus examination demonstrated sub-retinal haemorrhage and edema (Figure 1) whereas fluorescein angiography (FA) confirmed the presence of neovascular membrane (Figure 2).

The patient was treated with a total of three intravitreal anti-vascular endothelial growth factor (anti-VEGF) (aflibercept) injections at one-month intervals.

**Figure 3:**
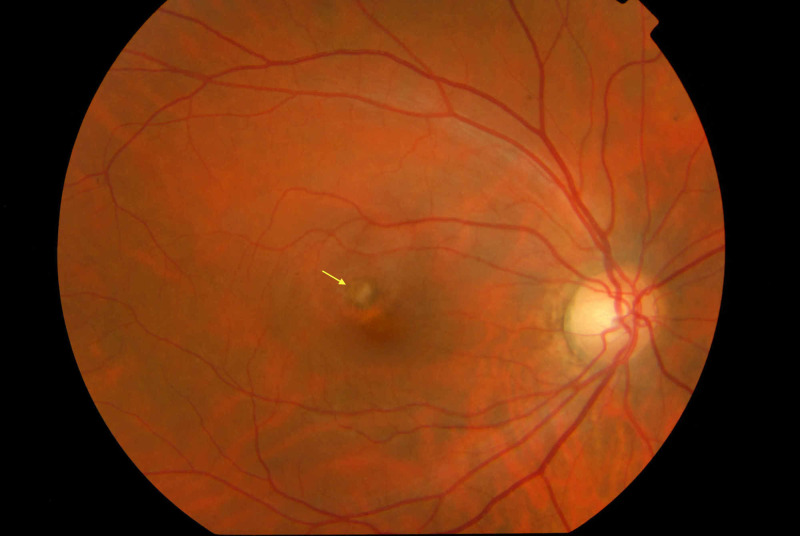
Fundus photography depicting chorioretinal scar (yellow arrow)

**Figure 4:**
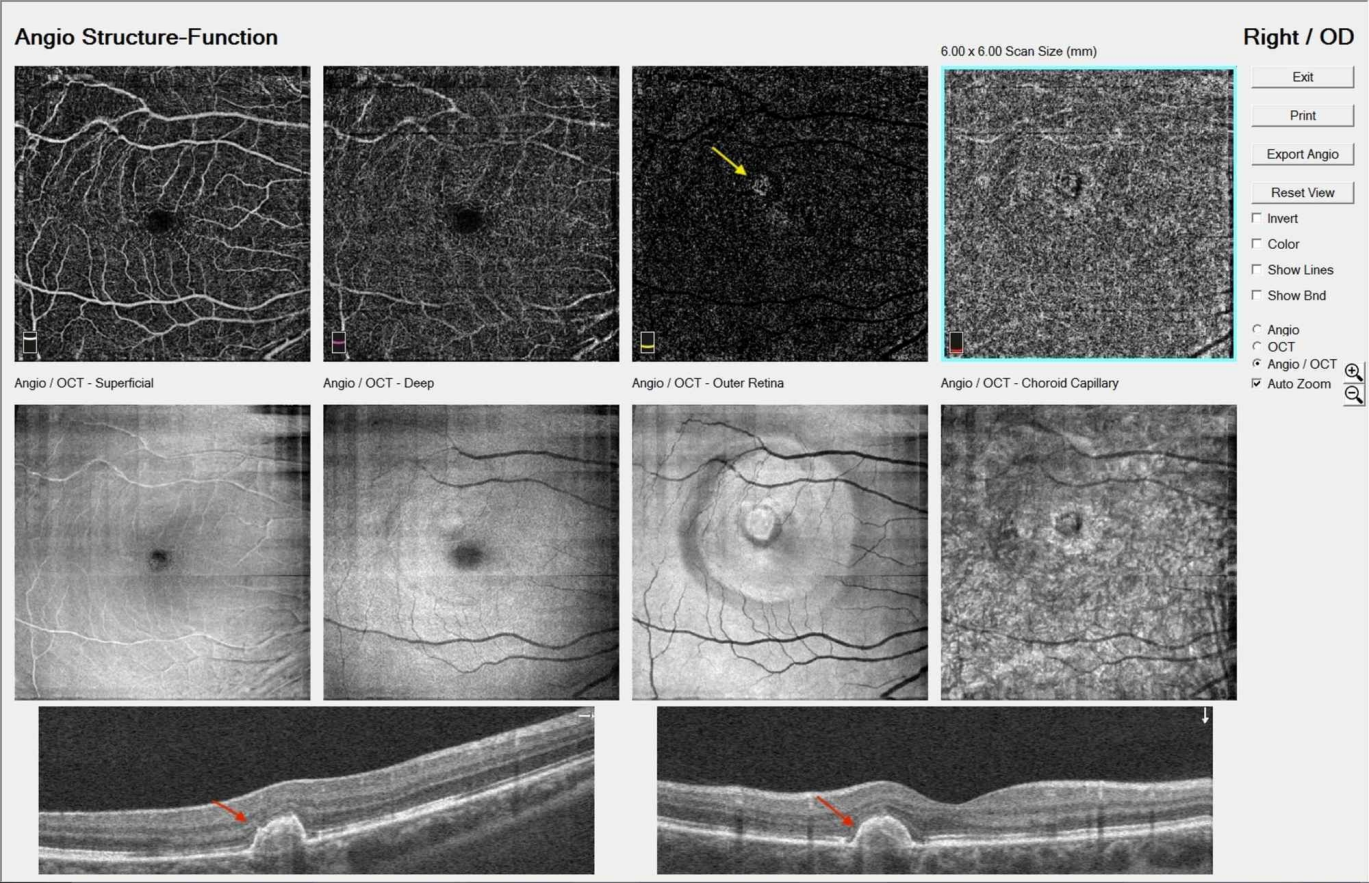
OCT and OCT-angiography depicting pigment epithelium detachment hypertrophy (red arrow) and the area of neovascular membrane (yellow arrow) (last follow-up examination) OCT: Optical coherence tomography

Follow-up examinations were conducted one day, one week, one month, three months and one year post injections. During the last follow-up examination, the patient’s BCVA was 20/20 and the fundus examination revealed a chorioretinal scar with peripheral hyperpigmentation, without the presence of hemorrhage nor edema (Figure 3). OCT demonstrated a pigment epithelium detachment with a hyperreflective area without the presence of sub-retinal fluid or retinal edema, while OCT angiography showed that the neovascular membrane remnants had no signs of activity (Figure 4).

## Discussion

The recent expansion of laser applications has led to increased prevalence of retinal injuries. The current treatment of laser-induced injuries is limited to systemic use of corticosteroids, while vitamin and antioxidant supplements may also be beneficial in reducing the risk of phototoxic damage to the retina and promoting recovery [5,6].

The visual outcome may vary according to the location of the injury. Chen et al. recently reported a retinal laser-induced burn associated with BCVA 20/20, despite the remarkable vitreous haemorrhage and retinal edema [7]. Another case report of a laser beam, however, which focused on the macula, caused a scar in the fovea with consequent central scotoma and BCVA 6/60 with no further improvement at an 18-month follow-up examination [8].

There are some interesting reports which highlight the persistence of focal disruptions of RPE and photoreceptor outer segment, with resultant RPE atrophy either by accidental Nd:YAG laser or by green laser pointer exposure [9,10]. There are also reports of choroidal neovascularization after laser injury that were successfully treated with photodynamic therapy [11]. Another case report also described anti-VEGF-mediated visual acuity increase in a patient with laser-induced sub-retinal haemorrhage [12].

In this case, the patient developed choroidal neovascular membrane a month and a half after alexandrite laser-induced injury. There are previous reports of retinal injuries and choroidal neovascularization secondary to alexandrite laser exposure. A patient developed choroidal neovascularization and sub-retinal haemorrhage which resolved after administration of five intravitreal ranibizumab injections and some others developed sub-retinal haemorrhage with associated sub-retinal fluid, which resolved after a single dose of intravitreal injection of bevacizumab 1.25 mg/0.05 ml [13,14].

Here, we applied anti-VEGF therapy (aflibercept), which is used in the treatment of many retinal diseases, such as diabetic macular edema, age-related macular degeneration and primary choroidal neovascularization. The choroidal neovascular membrane developed approximately six weeks after laser injury, findings which are consistent with the results of Ryan's study in which the neovascular membrane developed one to eight weeks after laser injury [15]. Despite the lack of clear guidance on the laser-induced secondary neovascularization, we used three intravitreal anti-VEGF injection at one-month intervals in order to decrease retinal edema and limit vascular permeability. This resulted in complete visual function improvement and no remarkable deterioration at the year follow-up examination after the injury. In addition, we highlight that this is the first report of using intravitreal aflibercept for the treatment of laser-induced choroidal neovascularization.

## Conclusions

Our report describes a case of secondary choroidal neovascularization associated with accidental retinal photic injury due to alexandrite laser during a hair removal procedure. Despite the patient’s functional recovery, this event illustrates the risk of developing irreversible visual problems due to exposure to lasers encountered in everyday life. The public should be aware and cautious of this potential hazard and the use of protective goggles should be strongly advised when handling or exposing oneself to current laser technologies.
